# Tonsillitis-Related Arthritis: Advanced Understandings of Tonsillitis and Sterile Inflammatory Arthritis

**DOI:** 10.1155/2021/2983267

**Published:** 2021-12-23

**Authors:** Shigeto Kobayashi, Issei Kida, Yuuki Makiyama, Yoshinori Taniguchi, Kurisu Tada, Naoto Tamura

**Affiliations:** ^1^Department of Internal Medicine and Rheumatology, Juntendo University Koshigaya Hospital, Koshigaya, Saitama 343-0032, Japan; ^2^Department of Otolaryngology, Yokohama City Minato Red Cross Hospital, Yokohama, Kanagawa 231-8682, Japan; ^3^Department of Endocrinology, Metabolism,Nephrology and Rheumatology, Kochi Medical School Hospital, Kochi University, Nankoku, Kochi 783-8505, Japan; ^4^Department of Internal Medicine and Rheumatology, Juntendo University School of Medicine, Tokyo 13-8421, Japan

## Abstract

A 49-year-old man developed acute aseptic arthritis of the nonmigratory and asymmetrical type in his knee, ankle, and bilateral metatarsal joints 13 days after treatment with antibiotics for acute tonsillitis. He was diagnosed with tonsillitis-related arthritis after other rheumatic diseases were ruled out. Treatment with salazosulfapyridine, methotrexate, and methylprednisolone for 3 months did not completely improve. Then, tonsillectomy was undertaken and arthritis rapidly improved. *Finegoldia magna* (previously *Peptostreptococcus magnus*) was cultured from the microabscesses of the resected tonsils. After outpatient follow-up, the patient did not experience a relapse of arthritis for more than 2.7 years without any treatment. Poststreptococcal reactive arthritis (PSRA) is well described. However, up to 40% of patients with tonsillitis-related arthritis did not demonstrate evidence of streptococcal infection. It is noted that tonsillectomy is necessary to remove the tonsillar microabscesses and eradicate bacterial infection of the tonsils, especially for patients with a prolonged and/or recurrent course of PSRA and/or tonsillitis-related arthritis.

## 1. Introduction

Reactive arthritis (ReA) is sterile arthritis that occurs in genetically predisposed individuals who have human leukocyte antigen- (HLA-) B27 secondary to an extra-articular infection, usually of the gastrointestinal or genitourinary tract. *Salmonella, Shigella, Yersinia,* and *Chlamydia* are the bacteria involved in this disease [1, 2]. On the other hand, sterile arthritis following group A *β*-hemolytic streptococcal tonsillitis or upper respiratory tract infection is described as poststreptococcal ReA (PSRA) [3, 4]. Compared with acute rheumatic fever (ARF), PSRA is characterized by a latent period of around 10 days between the antecedent streptococcal infection and the development of nonmigratory arthritis, which is shorter in 7–10 days than that in 10–28 days' period seen in ARF; longer duration of arthritis, which is more than 2 months longer than 2 to 3 weeks in ARF; poor response to aspirin; the absence of carditis; the marked severity of arthritis; and a tendency for recurrence and persistence of arthritis [3–6].

We previously reported on adult patients with PSRA and/or tonsillitis-related arthritis, some of whom were resistant to antibiotics and nonsteroidal anti-inflammatory drugs (NSAIDs) underwent tonsillectomy: viable bacteria in microabscesses from the resected palatine tonsils were demonstrated; moreover, arthritis completely disappeared by the removal of the infected tonsils [7, 8], and no one experienced a recurrence of arthritis. Herein, we describe the case of a patient with tonsillitis-related arthritis in whom streptococcal infection was not found and whose arthritis was resistant to NSAIDs, salazosulfapyridine (SASP), methotrexate (MTX), and glucocorticoid treatment for 3 months. Then, tonsillectomy was undertaken, and after removal of tonsillar microabscesses, his arthritis rapidly disappeared.

## 2. Case Presentation

A 49-year-old man was hospitalized at a local district for severe sore throat and fever (temperature, 40°C) that persisted for several days. He was diagnosed with acute tonsillitis and treated with antibiotics. The microbiological examination was not performed at that time before the administration of antibiotics, and elevated levels of anti-streptokinase (ASK) and anti-streptolysin O (ASO) antibodies were not observed at the hospital. On his 13th day of confinement, the patient rapidly developed arthritis in his right foot and left knee, and his C-reactive protein level increased to 10.13 mg/dl (normal, <0.3 mg/dl). His otorhinolaryngologist could not associate his tonsillitis with arthritis, and the patient read our article on tonsillitis-related arthritis. He then consulted an orthopedic doctor, who, in turn, referred him to our clinic for specialist assessment.

**Figure 1:**
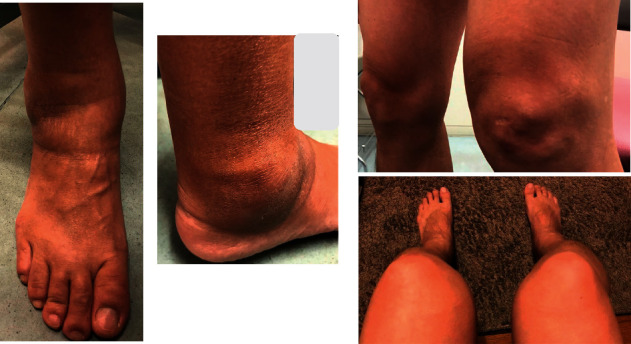
Case presentation. The patient presented with inflamed joints of the left ankle and right knee when he was referred to our hospital.

Upon presentation, the patient was walking very slowly on a cane, was afebrile, and had arthritis of both knees and both ankles, as well as the second to fourth metatarsal joints of both feet. Inflamed joints were found predominantly on his left knee and right ankle (Figure 1). A knee aspirate was completed: no crystal was found in the synovial fluid, and no bacteria were cultured.

The patient had no history of skin and eye involvement or gastrointestinal and urinary tract infections. He has had episodes of recurrent tonsillitis since he was 12 years old. His C-reactive protein level was 5.36 mg/dl. The serum level of uric acid was within the normal level. Rheumatoid factor and other autoantibodies were negative, as were ASK and ASO antibodies. Human lymphocyte antigen- (HLA-) B27 was not demonstrated.

**Figure 2:**
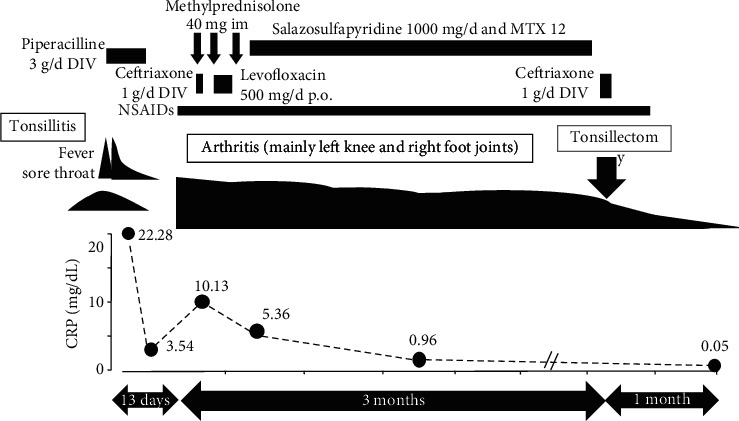
Clinical course. Since arthritis was not improved with the treatments for more than 3 months, tonsillectomy was undertaken. No arthritis was demonstrated 1 month after the surgery, and no recurrence of arthritis was found for more than 2.7 years without any treatment.

He was diagnosed with tonsillitis-related arthritis and recommended the removal of his palatine tonsils as written in our article. Although, the treatments of NSAIDs (SASP) (1 g/d, which is the maximum dose approved in Japan) and MTX (12 mg/week; maximum dose approved in Japan, 16 mg/week) for more than 3 months and intramuscular methylprednisolone (3 injections of 40 mg) did not completely improve the patient's arthritis. Therefore, tonsillectomy was undertaken, and *Finegoldia magna* (previously *Peptostreptococcus magnus*) was demonstrated in the resected tonsils. A slight contracture on the patient's left knee, but no arthritis, was found 1 month after the surgery as we expected. The patient did not experience a relapse of arthritis for more than 2.7 years without any medication (Figure 2).

## 3. Discussion

PSRA is widely understood to be distinct from acute rheumatic fever (ARF). Diagnostic criteria for PSRA include persistent, additive, nonmigratory acute arthritis in one or more joints, evidence of prior group A *β*-hemolytic streptococcal infection, and the lack of other major criteria for ARF [3–9]. However, it is not always easy to diagnose PSRA since it needed to be distinguished from viral arthritis, septic arthritis, and diseases of peripheral spondyloarthritis [4], and the spectrum of this disease is wide and significantly heterogeneous [10, 11].

In our old series of adult patients, only 62% of patients were positive for ASO and/or ASK, and only 57% of throat cultures for group A streptococcus were revealed to be positive [7, 8]. This may be due to the reason that some of our adult patients had a long period and recurrent history of chronic tonsillitis and subsequent arthritis [8]. Moreover, it was reported that 20–40% of patients were unable to demonstrate evidence of streptococcal infection [7, 8, 12]. *Pseudomonas aeruginosa, Klebsiella oxitosa, Chlamydia trachomatis, Staphylococcus, Serratia,* group B *Streptococcus, Staphylococcus aureus,* and *Peptostreptococcus* were isolated from throat swabs of our patients who were not demonstrated evidence of infection of *Streptococcus* [7, 11]. Our patient in this case report had a history of recurrent tonsillitis, and it may be possible that *Streptococcus* was replaced by other bacteria such as *F. magna* which are resistant to antibiotics and/or phagocytosis.

Therefore, clinicians should understand that not only *Streptococcus* but also other bacteria will induce sterile arthritis similar to that of PSRA, although its clinical manifestations do not differ from those of PSRA. In addition, many bacteria have been reported to be involved in a disorder of “widely interpreted ReA” [13]. Since evidence of streptococcal infection was not found in the case described herein, we prefer to use the term “tonsillitis-related arthritis” in diagnosis but not PSRA, based on the definition of the criteria proposed by the Fourth International Workshop of Reactive Arthritis in 1999 [14]. The term “ReA” could be used only if the clinical picture and the microbes involved are associated with HLA-B27 and spondyloarthritis [8, 14].

Most patients, especially children with PSRA or tonsillitis-related arthritis, respond well to antibiotics and NSAIDs and resolve within 8 weeks [9]. However, it is noted that 77% of adult patients with PSRA did not resolve arthritis within 6 weeks [11]. The bacteria involved in classical ReA reside intracellularly in host cells. However, *Streptococcus* and *F. magna* are extracellular bacteria, some of which can produce biofilm patches around them that are capable of not only escaping contact with antibiotics but also avoiding phagocytosis and/or autophagy inside the macrophages/monocytes [15]. Therefore, it is essential to understand that microabscesses existing inside the palatine tonsils are the real foci of bacterial infection, especially in patients who are resistant to antibiotics. In our old study, the mean duration of arthritis before diagnosis was much longer in 8 patients (38.6 ± 47.0 months; mean ± standard deviation) who had undertaken tonsillectomy than that of 13 patients (13.0 ± 17.9 months) who were not undertaking tonsillectomy (data not shown) [8, 16].

Moreover, tonsils have been widely understood as the pathogen reservoir, which indicates that infection of the tonsils has multiple foci, the microbiome is polymicrobial, and bacteria are present throughout the tissue [15]. Therefore, the persistence of viable *Streptococcus* and other extracellular bacteria in the CD14+ cells of the tonsils would allow for their movement to the joints and induce aseptic arthritis as we previously demonstrated the fragments of *Streptococcus* in a patient's synovial fluid cells [7].

Therefore, viable bacteria persistently exist in the tonsils for a long period, and this might be an explanation why frequent recurrence and/or long-period treatment were experienced in adult patients with PSRA/tonsillitis-related arthritis [7, 8, 11].

## 4. Conclusions

Our case demonstrates that tonsillectomy is effective for the treatment of tonsillitis-related arthritis to eradicate the bacterial infection in the tonsils. If arthritis does not respond to prolonged treatment with antibiotics and NSAIDs and if, especially, patients have a history of recurrent tonsillitis, tonsillectomy is recommended for preventing comorbidities such as toxic shock syndrome [8, 17]. General practitioners, especially otorhinolaryngologists, orthopedic doctors, and rheumatologists, should be well aware of this disorder to diagnose appropriately and treat it efficiently.

## Data Availability

The datasets generated during and/or analysed during the current study are not publicly available but are available from the corresponding author on reasonable request.
